# Energy self-sufficient households with photovoltaics and electric vehicles are feasible in temperate climate

**DOI:** 10.1371/journal.pone.0227368

**Published:** 2020-03-04

**Authors:** Ursin Gstöhl, Stefan Pfenninger

**Affiliations:** Climate Policy Group, Institute for Environmental Decisions, ETH Zürich, Switzerland; Nanyang Technological University, SINGAPORE

## Abstract

The idea that households produce and consume their own energy, that is, energy self-sufficiency at a very local level, captures the popular imagination and commands political support across parts of Europe. This paper investigates the technical and economic feasibility of household energy self-sufficiency in Switzerland, which can be seen as representative for other regions with a temperate climate, by 2050. We compare sixteen cases that vary across four dimensions: household type, building type, electricity demand reduction, and passenger vehicle use patterns. We assume that photovoltaic (PV) electricity supplies all energy, which implies a complete shift away from fossil fuel based heating and internal combustion engine vehicles. Two energy storage technologies are considered: short-term storage in lithium-ion batteries and long-term storage with hydrogen, requiring an electrolyzer, storage tank, and a fuel cell for electricity conversion. We examine technological feasibility and total system costs for self-sufficient households compared to base cases that rely on fossil fuels and the existing power grid. PV efficiency and available rooftop/facade area are most critical with respect to the overall energy balance. Single-family dwellings with profound electricity demand reduction and urban mobility patterns achieve self-sufficiency most easily. Multi-family buildings with conventional electricity demand and rural mobility patterns can only be self-sufficient if PV efficiency increases, and all of the roof plus most of the facade can be covered with PV. All self-sufficient cases are technically feasible but more expensive than fully electrified grid-connected cases. Self-sufficiency may even become cost-competitive in some cases depending on storage and fossil fuel prices. Thus, if political measures improve their financial attractiveness or individuals decide to shoulder the necessary investments, self-sufficient buildings may start to become increasingly prevalent.

## Introduction

Climate change mitigation requires eliminating fossil fuel emissions from the energy sector. Solar photovoltaics (PV) is not only one of the most promising technology options to play a major role in a clean energy system, but also opens up the possibility for a much more decentralized supply of electricity. It has led to the idea of energy self-sufficiency or energy autarky at scales ranging from individual buildings to larger political regions [1]. However, maintaining a secure and affordable supply of energy remains an important aspect of the energy transition. Energy self-sufficiency is primarily a political goal that requires technical solutions like long-term storage for its actual implementation, the feasibility and costs of which are still unclear [2]. Here we investigate the technical and economic feasibility of energy-independent households relying on PV electricity for the case of Switzerland, which can be seen as representative for the situation in a temperate and highly industrialized country. The novelty of this study is that we use a single, integrated approach to investigate a range of building types and demand scenarios, while fully considering mobility and heating demand as part of the building’s self-sufficiency.

Switzerland is party to the Paris Agreement which has the main aim of limiting global temperature increase above pre-industrial levels to 2°C [3]. The Swiss government has furthermore adopted a net zero emissions target by 2050 [4]. Switzerland has also decided to phase out nuclear power following the Fukushima disaster, and the last nuclear power plant will have to shut down by 2034 [5]. In 2017, 31.7% of Swiss electricity came from nuclear plants, with the majority of the remainder from hydropower (59.6%) and only 4.7% from conventional thermal plants, and 4.0% from non-hydro renewables [6]. Since hydropower has only limited additional potential, replacing the lost nuclear generation with other emissions-free electricity sources will be a major challenge, particularly as electricity supply rises along with electrification of heating and transportation [7]. Increasing decentralized production by households is an attractive option in this context: the residential PV potential has been estimated as between 11 to 19 TWh in Switzerland in 2050 [8]. If electric mobility becomes increasingly common, as we should assume will be the case, household electricity use will increasingly include charging of vehicles [9]. Thus, the question of decentralized and potentially self-sufficient electricity generation based on large-scale PV deployment combined with the large-scale deployment of electric mobility is particularly relevant, not just for Switzerland, but for any highly industrialized economy in a temperate climate facing a similar clean energy transition challenge.

Energy independent residential dwellings have been addressed in the literature, as shown in the overview in Table 1. However, past studies were often constrained to special conditions, limiting their general applicability. Some of the studies outlined in the table help us build a comprehensive picture of how individual components in a self-sufficient household work, e.g. by optimizing combined PV and battery deployments [10–13]. Other studies analyze individual cases of net zero-energy buildings [14, 15], but these studies do not include considerations of electrified mobility and how that would affect building-level self-sufficiency. There are no studies with a view of the bigger picture that make more general statements about the potential for residential self-sufficiency in a country like Switzerland. The more systems-oriented studies are based on the concept of decentralized energy systems, where individual units are still connected to a local distribution network and the study aim is to design optimal local energy systems (e.g. [12], [16]). In these systems, energy is supplied primarily through renewables and converted and stored in a range of different energy carriers.

**Table 1:** Overview of key past studies on residential energy self-sufficiency [10–19].

Source	Aim and methods	Results	Differences to our study
G**ROSSPIETSCH**, Thommes (10)	Simulation model which shows how, when, and where a self-sufficient neighborhood is economically feasible.	How: Through solar PV together with a battery system (1) or together with batteries and hydrogen storage (2). When: Costs have to decrease 21% (1) and 51% (2), respectively, to be competitive to households relying on fossil fuels. Where: Little seasonality (1) to high seasonality (2).	• No timeframe • Ranging from low to high latitudes • Neighborhood containing a community of households of different sizes and an office building • No sensitivity analysis of factors • Technically not feasible with current efficiencies • No consideration of electric mobility
Murray, Orehounig (11)	The potential of long-term and short-term storage systems are modelled in a decentralized neighborhood with minimizing costs and CO_2_-emissions. Three scenarios are created and modelled until 2050. The model is tested on two sample municipalities in Switzerland.	Modelled data regarding technologies and price developments until 2050. Development in the locations of Zernez and Altstetten, Switzerland, are presented. Furthermore, the importance of retrofitting the current building stock is mentioned because of the limitation of renewable energy.	• No information about single buildings is given • Focus on the two case study locations • Local gas, electricity, and heating grid are present and used • No consideration of electric mobility
Zhang, Campana (12)	Grid-connected PV-hydrogen/battery systems are investigated with the creation of three strategies for the hydrogen storage. Moreover, a high and a low-cost scenario are created.	Strategies to produce and store hydrogen are dependent on the electricity price and make sense when a seasonal mismatch between load and PV production occurs. Hydrogen storage shows a better performance when grid power fluctuation is considered.	• Connected to the grid • No clear relation to the residential sector • No information about sizing the hydrogen or battery system
Weniger, Tjaden (13)	Optimal sizing of PV-battery-systems in the residential sector are analyzed. In addition, a sensitivity analysis is conducted regarding PV and battery size in different cost scenarios.	The absolute yield and the PV generator orientation will be of small relevance in the future. The most optimal PV sizes are reached in small-scale systems with high self-consumption rates and degree of self-sufficiency (above 70%). In a long-term perspective, PV systems with batteries will be the most economical solution.	• Connected to the grid • Special case (single-family building) located in Lindenberg, Germany • Information about the household is restricted to its electricity demand of 4 MWh/year • No renewable heating system is considered • No hydrogen production is considered • Feed-in tariff is applied in the modelling • No consideration of electric mobility
Good, Andresen (14)	Solar energy solutions are modelled and compared with the aim of fulfilling the requirements of a net zero energy balance.	If the building only uses solar PV, the net zero energy balance shows the better performance than using solar thermal collectors in combination with PV or using the technology of hybrid photovoltaic-thermal modules.	• Special case of a single-family building in Norway • Connected to the grid • No storage of electricity • No consideration of electric mobility
Milan, Bojesen (15)	A model is developed to optimally size a 100% renewable supply system while considering overall costs. It is applied on a case study in Denmark.	The requirement of a net zero-energy building is successfully applied and optimally if the PV system is combined with a heat pump. Available rooftop area is not used for solar thermal collectors.	• Connected to the grid • Special case with conditions of Northern Denmark • No hydrogen production is considered • No consideration of electric mobility
Maroufmashat, Fowler (16)	A generic mathematical model is developed to manage future communities which use hydrogen as an energy carrier. The model is applied to a case study consisting of four energy hubs which work together.	Results are based on a case study located in Ontario, Canada. Annual total emissions and the levelized costs of hydrogen of the energy hub networks are presented. To produce hydrogen on-site is more economical than purchase it. The environmental advantages of hydrogen vehicles are assessed in addition.	• Focus on hydrogen production only • A network of energy hubs is investigated with no further explanation of their composition • No detailed information about the residential sector • No storage of electricity in batteries • Consideration of hydrogen fueled cars
Lang, Ammann (17)	The creation of a model to simulate the performance of rooftop PV for different building types to assess the techno-economic potential of PV self-consumption.	In the small residential sector, 40% of the produced electricity is self-consumed. In the large residential sector 80%. Furthermore, the internal rate of return is calculated to measure profitability.	• The aim is not to be self-sufficient • No facade PV • Connected to the grid • No storage of electricity
Prognos (18)	The aim is to build a quantitative basis for political and societal discussions. This is done by modelling three scenarios to investigate the effect of framework conditions, influencing factors, political interventions, and climate and energy goals.	Quantitative data of the three scenarios until 2050.	• Modelling of the entire Swiss energy landscape • No statement regarding self-sufficiency • Diverse technologies are modelled
SFOE (19)	Different heating systems are simulated with the aim to find the most efficient combination of heat pumps and solar energy solutions.	Different advantages and disadvantages are mentioned. The most economical system is composed of an air-to-water heat pump and solar PV.	• Special case of a single-family building with a fixed energy consumption • Only heating system is of interest • No storage of electricity

To synthesize past work into an overall understanding of the conditions under which household self-sufficiency based on PV electricity is possible, we build generic cases that vary on the dimensions of household type, buildings type, electricity demand, and electric mobility behavior, and build scenarios for their household energy demand by 2050. To construct these cases, we draw on data and make assumptions based on the detailed studies shown in Table 1. We investigate three questions:

- In which of these cases, if any, are households able to satisfy their energy demand with self-produced electricity?
- Which factors influence the technological and economic feasibility of self-sufficiency?
- How would energy costs for self-sufficient households compare to those still relying on fossil fuels and grid-based electricity by 2050?

The more densely populated an area, the more difficult it will be to generate enough electricity for that area with solar PV only. Single-family buildings therefore achieve self-sufficiency most easily. However, with technological improvement leading to more efficient PV panels, even some multi-family buildings can be self-sufficient. In all cases, cost driven by storage needs is a key issue, and implementing fully built-out rooftop PV on fully electrified buildings while remaining connected to the grid is the most cost-attractive solution under almost all assumptions.

## Methods

### Definition of cases

**Fig 1:**
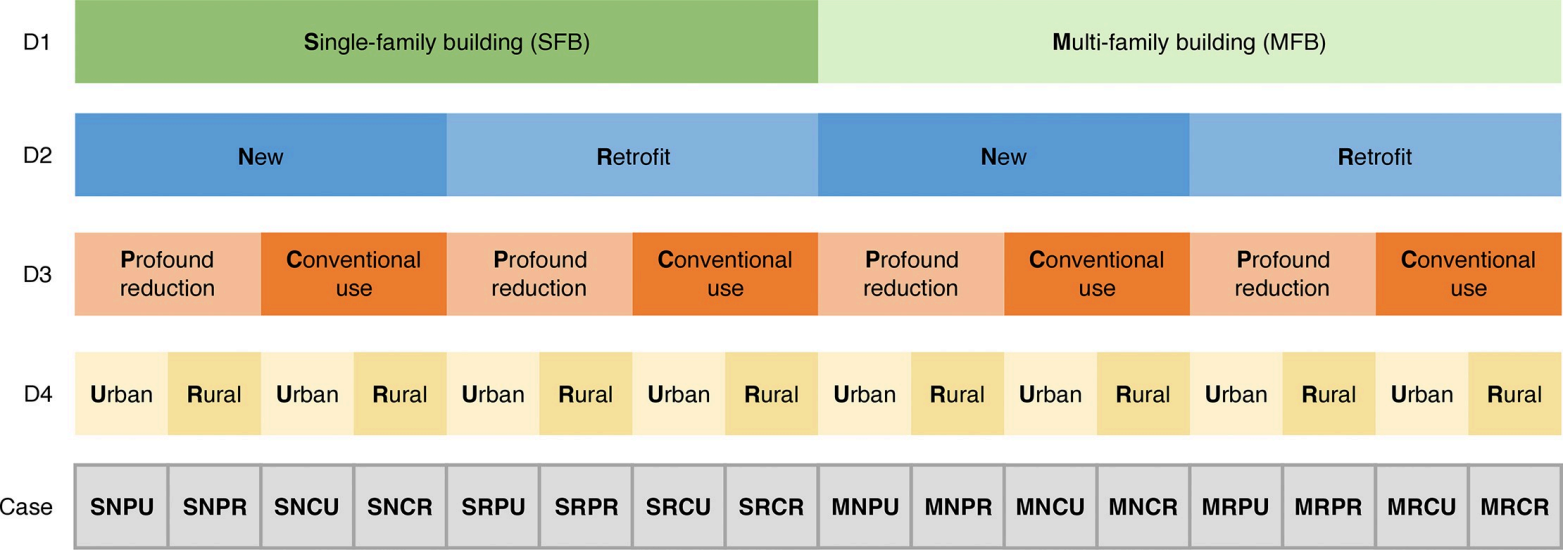
Outline definition of the sixteen self-sufficient cases with abbreviations. D1 (green) represents the first dimension of household types, D2 (blue) represents the second dimension of building types, D3 (orange) represents the third dimension of projected electricity demand, D4 (yellow) represents the fourth dimension of BEVs and spatial behavior. Each case is coded with an abbreviation. First letter: S = SFB, M = MFB; Second letter: N = New building, R = Retrofitted building; Third letter: P = Profound electricity reduction, C = Conventional electricity use; Fourth letter: R = Rural area, U = Urban area.

We define sixteen cases through which we examine energy self-sufficiency, aiming to represent expected state-of-the-art technologies available by 2050. The cases are constructed by varying four dimensions which each represent a binary choice on key assumptions for stylized housing types, as shown in Fig 1.

Dimension 1 (household type) defines energy requirements of single-family households (SFHs) in single-family buildings (SFBs) and multi-family households (MFHs) in multi-family buildings (MFBs). Dimension 2 (building type) distinguishes between new and retrofitted buildings, with a focus on their differing energy requirements for space heating. Dimension 3 (electricity demand) is defined by behavioral changes and technology developments which may lead to lower energy demand. Because future electricity demand cannot be predicted we differentiate between two stylized cases: a profound electricity reduction and a scenario with a conventional electricity use. This only affects electricity demand not already included in space heating and electric mobility (covered by dimensions 2 and 4, respectively). Dimension 4 assumes a complete electrification of passenger cars in both cases, with differentiation of mobility behavior based on a typical urban or rural household. This is an important dimension because driving behavior and developments in the car industry will profoundly influence electricity demand [20–22]. We only consider battery electric vehicles (BEVs) here. Fuel cell electric vehicles (FCEVs) can be seen as an alternative provider of electric mobility, but we do not explicitly consider them, instead assuming that their usage patterns and resulting electricity demand would be similar as those for BEVs.

Table 2 summarizes all assumptions used to quantify the four dimensions. Many of the future trends are taken from the “New Energy Policy” (NEP) scenario from the Swiss Energy Perspectives 2050 created on behalf of the Swiss Federal Office of Energy (SFOE) by Prognos (18).

**Table 2:** Assumptions for all cases. Dimension 1 determines the dimensioning of SFBs and MFBs with their electricity consumption and domestic hot water (DHW) demand. Dimension 2 determines usable rooftop share and yearly space heating energy demand of newly built SFHs and MFHs today and in 2050 with the individual reduction potential and the projected SPF of air-to-water heat pumps in 2050. Dimension 3 determines changing electricity demand with the consideration of cooling and lighting demand, and dimension 4 considers the required electricity for transportation, per house unit and month, derived by estimating in passenger kilometers per person in both a rural and urban setting.

All cases	Overall assumptions				Sources
Inverter efficiency	99%				[23]
Performance ratio	0.9				[24]
Solar irradiation, rooftop	1,000 kWh/m^2^/year				[25]
Solar irradiation, facade	800 kWh/m^2^/year				[10], [26], [25]
**Dimension 1: building type**	**SFBs**		**MFBs**		
Occupancy rate per household	4		4		[10], [27]
Occupancy rate per building	4		20		[10]
Stories	3		5		[10], own assumptions
Floor area	200 m^2^		900 m^2^		[10], [28], [29], [30], [31]
Rooftop slope	35°		35°		[32], [33]
Rooftop area	81.4 m^2^		219.7 m^2^		Own assumptions
Facade area for PV production (33% of one facade side)	25.5 m^2^		67.7 m^2^		Own assumptions
Current electricity consumption of each house unit	5,200 kWh/year		19,250 kWh/year		[34]
DHW demand in 2050	10.9 kWh/m^2^; -26% compared to 2020 [18]		12.6 kWh/m^2^; -26% compared to 2020 [18]		[14], [19], [29], [30], [31], [35]
DHW efficiency factor in 2050	3.5; +22% compared to 2020 [18]		3.5; +22% compared to 2020 [18]		[18], [36]
**Dimension 2: building age**	**New buildings**		**Retrofitted buildings**		
Usable rooftop area	100%		70%		[37]
Yearly energy demand of SFHs–now	16.0 kWh/m^2^		22.5 kWh/m^2^		[14], [19], [29], [30], [31], [35], [38]
Reduction potential	-53%		-56%		[18]
Yearly energy demand of SFHs–in 2050	7.5 kWh/m^2^		9.9 kWh/m^2^		[14], [18], [19], [29], [30], [31], [35], [38]
Yearly energy demand of MFHs–now	18.2 kWh/m^2^		20.9 kWh/m^2^		[14], [19], [29], [30], [31], [35], [38]
Reduction Potential	-34%		-39%		[18]
Yearly energy demand of MFHs–in 2050	12.0 kWh/m^2^		12.7 kWh/m^2^		[14], [18], [19], [29], [30], [31], [35], [38]
SPF of air-to-water heat pumps–in 2050	4.8; +46% compared to 2018 [18]		4.4; -9% compared to new buildings [39]		[18], [36], [39], [40], [41]
**Dimension 3: demand**	**Profound reduction**		**Conventional use**		
Decrease of electricity reduction compared to [34]	-29%		-7%		[18]
Climate change impact on cooling electricity demand	+2.5% of today’s electricity consumption [34]		+2.5% of today’s electricity consumption [34]		[42], [43]
Share of lighting of the entire electricity demand	13.3%		13.3%		[38]
**Dimension 4: mobility**	**Urban SFBs**	**Rural SFBs**	**Urban MFBs**	**Rural MFBs**	
Passenger km (pkm) in 2050 per person times occupancy rate	4,814	7,554	4,201	6,439	[44], [45]
Pkm in 2050 per house unit	19,255	30,215	84,021	128,785	[45], [46]
Required kWh/100 km in 2050	12	12	12	12	[47], [48], [49]
Required kWh in 2050 per house unit	2,311	3,626	10,082	15,454	Calculation based on above sources
Required kWh in 2050 per house unit and month	193	302	840	1,288	Calculation based on above sources

Each of the cases is modelled for a self-sufficient and “alternative” future. The self-sufficient cases assume full electrification of mobility and heating demand. The alternative cases assume that household energy is drawn from the electricity and gas grids, as well as the continued use of internal combustion engine (ICE) vehicles. We assume an equal purchasing price and lifetime of BEVs and ICEs by 2050, and the cost of vehicle purchase is not further included in our cost calculations. The use of EV batteries for additional balancing in households is not considered given the uncertainty about EV availability based on different mobility profiles. To compare the cases of fully self-sufficient electrification with a grid-connected full electrification scenario, we also investigate a third future where buildings remain connected to the grid. These cases are designed with or without on-site battery storage (in battery cases, all of the electricity is assumed to pass the battery), and with rooftop PV efficiencies of 22.1% and 27.2%. Two market scenarios are used. Under a scarce market, electricity purchasing price is 0.31 CHF/kWh (+20% of the 2050 assumption from [11]) and a selling price half this purchasing price. In an abundant market the purchasing price is of 0.21 CHF/kWh and no electricity can be sold back to the grid. All PV oversupply is sold, and in the no-battery case, all demand has to be satisfied at market prices. This third future is used solely to compare the cost of grid-connected electrification with fully self-sufficient electrification.

The key assumptions relate to the area available for PV systems, demand which needs to be met by this PV capacity, and the availability of storage.

#### PV potential

To determine the available solar electricity production, each case was optimized for an optimal solar PV area to meet its demand, by applying a generalized reduced gradient descent algorithm as implemented in the Microsoft Excel Solver add-in (see S2 and S3 Data). The monthly electricity balance was calculated for each case and PV cell efficiency by subtracting the electricity demand from the produced electricity. The algorithm computes the optimal area of solar PV, considering a surplus production for hydrogen storage, and by taking restrictions on the rooftop and facade sizes into account. An optimal solar PV area can be reached when the energy balance is exactly equilibrated at zero. The objective is to produce a sufficient amount of energy with the given parameters of Table 2 while minimizing the panel area, which reduces costs. Additionally, an increase in PV efficiency enables a smaller area to cover for the same output. Rooftop and facade PV electricity production is unevenly distributed over the year. Average monthly production was determined through the online calculator tool of EnergieSchweiz (33) for ten locations in Switzerland (1072 Forel, 2942 Alle, 3076 Worb, 3930 Visp, 5076 Bözen, 6020 Emmen, 6952 Canobbio, 7000 Chur, 8046 Altstetten, 8853 Lachen). The tool uses long-term solar irradiance data for the specific location, as well as installed module capacity, tilt and azimuth angles, to compute long-term average monthly electricity generation. Panels were assumed to be fixed with a tilt angle according to the rooftop slope given in Table 2 and receive a monthly irradiance share calculated based on the average of a south-oriented, west-oriented, east-oriented panel. The same approach was used for facade PV systems. Data for the feasibility assessment with a realistic generation profile from Renewables.ninja are described further below.

#### Demand

The energy demand for each case is quantified on a monthly basis to capture the fact that lighting and space heating demand vary seasonally [38]. Regarding dimension 1, the household types need a specific size, which we base on adjusted data from measurements performed by Grosspietsch, Thommes (10). Annual energy demand of SFHs and MFBs for domestic hot water (DHW) was calculated by taking the mean of reported future demand from the sources given in Table 2, in order to arrive at a robust estimate of potential demand reductions. Space heating demand is not only influenced by the household type but also by the building type. The sources given in the table were used to derive an average value for new and retrofitted SFBs and MFBs [29–31]. We assume an air-to-water heat pump for space heating based on assessing different advantages and disadvantages of several possible heating systems [18, 19, 40, 50]. As for mobility demand, we assume that buildings in an urban setting require less vehicle range than buildings in a rural setting. This is based on data of the Swiss Federal Statistical Office (FSO) [45] and the Swiss Federal Office for Spatial Development [44, 46]. We also assume an occupancy rate in cars of 1.6 in 2050 based on the fluctuating trend of the past described by the [45].

#### Storage

In periods where PV-generated electricity cannot cover demand, residents will have to rely on stored energy. Short-term storage can be covered by lithium-ion batteries. Lorenzi and Silva [51] state that efficiencies of 92% were already achieved in 2016. Hence, we assume 92% to be a solid value also for 2050. Moreover, the discharge depth is almost 100% and the monthly self-discharge rate is estimated between 1% and 5% [52, 53]. Therefore, we assume the discharge depth and the monthly self-discharge rate to be negligible in 2050, in particular given that the systems are in use on a daily basis. A further assumption is that all produced energy has to pass the battery system. Battery dimensioning is assessed for each case separately with a battery storage capacity twice as large as the average daily energy demand in kWh. This size is chosen as Tesla [54] suggests that a battery should be able to cover one full day of energy usage, while Umweltarena [55] recommends a battery which covers up three days. Long-term storage is based on hydrogen production and reconversion into electricity. The processes of electrolysis, compression and fuel cell usage require more steps than electricity storage in batteries. PEM electrolyzers have an efficiency of 70% [56] in 2017 and projections reach efficiencies of up to 85% [57] or higher [58] in 2030. Compression is assumed to be integrated in the electrolyzer with a pressure of 30 bar [59]. An electrolyzer efficiency of 80% is thus reasonable in 2050. The efficiency of PEM fuel cells is provided by various sources [10, 49, 60]. Based on positive efficiency estimations by Miotti, Hofer (49) of 52% for 2030, we assume this value to be reached in 2050. We assume that electricity produced in the fuel cell also passes through the battery system. All of these steps result in an overall long-term storage efficiency of 38.3%. For each case, the size of the hydrogen tank is dimensioned to guarantee sufficient storage for hydrogen with an energy content equivalent to 100% of the building’s yearly end-use demand of electricity. The term *end-use demand* is defined as the energy demanded by users and should not be mixed up with the term *final energy consumption* which represents the amount of energy input needed to reach the end-use demand [38].

### Costs

If the self-sufficient cases are cost-competitive compared to similar cases still relying on fossil fuels and electricity from the grid, they could be attractive investments for households. To examine whether this might be the case, we calculate total discounted costs for each case:

$$Total\;costs\;\left[\frac{CHF}{kWh}\right]=\sum_{t=1}^{T}\frac{{{investment\;costs}_{t}+O\&M\;costs}_{t}}{{\left(1+i\right)}^{t}}$$ (1)

Variable *T* indicates the observed time horizon in both equations. *t = 1* is the year 2050. We assume a time horizon of 20 years and set the discount rate *i* to 4% in 2050, which represents the contemporary average discount rate of residential consumers [61]. Eq 4 is based on the LCOE definition of Hernandez-Moro and Martinez-Duart [62] and comprises investment costs (capital costs and balance-of-system (BOS) costs) and operation and maintenance (O&M) costs. BOS costs are defined as all costs occurring in the purchasing period minus the capital costs of a product. The BOS costs of a PV plant include, for instance, the costs of wiring, the inverter, planning, installation, and the transformer. O&M costs occur every year because of services to keep the technology in operation. Efficiency improvements of electrolyzers and fuel cells are included in the equations in Table 3 by assuming a cost reduction of 33.3% for electrolyzers and of 66.6% for fuel cells by 2050. The capacity of electrolyzers and fuel cells are adjusted to data of a multi-family zero-energy building in Brütten, Zurich [63], with 4.8 kW in SFBs and 9.7 kW in MFBs for electrolyzers, and 2.1 kW in SFBs and 4.1 kW in MFBs for the fuel cells. Furthermore, batteries have to be replaced after operating for 15 years. Cost data used for the self-sufficient and fossil/grid alternative cases are shown in Table 3 and Table 4, respectively. Where necessary, currencies are adapted to inflation and converted to 2019 CHF.

**Table 3:** Cost assumptions for the self-sufficient cases. All costs are in 2019 CHF.

	Price in 2050 [CHF_2019_]	Source	Lifetime [years]	Source
***Solar PV***				
*Investment costs [CHF/m*^*2*^*]*	200.-	[11], [64], own assumptions	25	[11]
*Investment costs [CHF/kWp] for 22*.*1% efficiency*	905.-	-	-	-
*Investment costs [CHF/kWp] for 27*.*2% efficiency*	735.-	-	-	-
*O&M costs [CHF/kWh]*	0.02	[11], [65], own assumptions	-	-
***Heat pump***				
*Investment costs–SFB*	36,000.-	[66]	20	[11]
*Investment costs–MFB*	136,800.-	Own assumptions	-	-
*O&M costs*	200.-	[66]	-	-
***Battery***				
*Capital costs [CHF/kWh]*	265.-	[48], [54], [67], own assumptions	15	[53], [54], [61], own assumptions
*BOS costs*	2000.-	[68]	-	-
*Additional Hardware costs (part of investment cost)*	740.-	[54]	15	[53], [54], [61], own assumptions
*O&M costs*	4.5% of investment costs	[61]	-	-
***Electrolyzer***				
*Capital costs [CHF/kW]*	$7^{\prime }633\;[CHF]*X^{-0.276}[kW]*\frac{2}{3}$	[69], [70], own assumptions	22	[11], own assumptions
*BOS costs*	30% of capital costs	[61]	-	-
*O&M costs*	5% of capital costs	[61]	-	-
***Storage tank***				
*Investment costs*	$600\;\left[\frac{CHF}{m^{3}}\right]*X_{CaseX}\;[m^{3}]$	[71], own assumptions	22	[11]
*BOS costs*	2500	Own assumptions	-	-
*O&M costs*	3% of investment costs	[61]	-	-
***Fuel cells***				
*Capital costs [CHF/kW]*	$6^{\prime }506\;[CHF]*Y^{-0.177}[kW]*\frac{1}{3}$	[11], [61], own assumptions	22	[11], own assumptions
*BOS costs*	30% of capital costs	[61]	-	-
*O&M costs*	10% of capital costs	[61]	-	-

**Table 4:** Cost assumptions for the alternative cases exchanging energy with the grid and using fossil fuels. All costs are in 2019 CHF.

	Price in 2050 [CHF_2019_]	Source	Lifetime [years]	Source
***Gas heating system***				
*New installation—SFH*	20,000.-	[72]	20	[11]
*Replacement—SFH*	14,000.-	[72]	20	[11]
*New installation—MFH*	76,000.-	[72], own assumptions	20	[11]
*Replacement—MFH*	70,000.-	[72], own assumptions	20	[11]
*O&M costs [CHF/year]*	650.-	[72]	-	-
***Air conditioning***				
*Price–SFB*	6000.-	[73]	20	Own Assumptions
*Price–MFB*	10,000.-	[73]	20	Own Assumptions
*O&M costs [CHF/system]*	150.-	[73]	-	-
***Energy prices***			**Change 2020–2050**	
*Electricity price [CHF/kWh]*	0.262	[11]	+24%	[18]
*Gas price [CHF/kWh]*	0.175	[18]	+43%	[18]
Gasoline price* *[CHF/kWh]*	0.29	[18]	+29%	[18]

* with a density of 0.775 kg/l and a heating value of 11.6 kWh/kg

### Sensitivity analyses

To test the feasibility of the self-sufficient cases in a more realistic environment, we consider daily data of a real location in addition to average monthly data. We assess the average of a south-oriented, an east-oriented, and a west-oriented rooftop in Worb, a municipality located in the Swiss Plateau region, for 2000 to 2015 using the PV simulation model provided by Renewables.ninja [74]. Renewables.ninja uses the GSEE simulation software [75], and was set up to compute hourly solar electricity generation based on module capacity, tilt and azimuth angle, and bias-corrected satellite-derived irradiance data from CMSAF SARAH, then averaging hourly to daily values. This location was chosen because of its position in the Swiss Plateau where most of the Swiss population is domiciled [76]. In a first step, Renewables.ninja provided the average daily output for the capacity of one kWp installed capacity of solar PV on a specific day (*Q*). Running a simulation for south-oriented (*Q*_*south*_), west oriented (*Q*_*west*_), and east-oriented (*Q*_*east*_) rooftops allows computation of the average daily capacity with one kWp of solar PV installed. To test the robustness of the cases in a generic environment, we normalize the yield data from Worb to 1,000 kWh/m^2^ by applying a 10.8% reduction to its 1,122 kWh/m^2^ annual average. The risk of not being able to provide enough energy is hedged by enforcing a hydrogen energy overproduction 1.75 times the found optimal value of production. The individual capacities per case (*L*_*CaseX*_) are calculated based on the knowledge of required needed rooftop and facade area. The output (*C*_*CaseX*_) in kWh/day is this performance multiplied by 24 hours. Eq 1 shows the entire approach:

$$C_{caseX}\;\left[\frac{kWh}{day}\right]=\frac{{(Q}_{south}+Q_{west}+Q_{east})}{3}*\left[\frac{kW}{kWp}\right]*(1-0.108)*L_{CaseX}\;\left[kWp\right]*24\;\left[\frac{h}{day}\right]$$ (2)

The necessary yield to cover energy demand on each day (*D*_*CaseX*_) is computed by dividing the optimized monthly energy production (*Y*_*MonthZ*_), which is required to satisfy the demand in this month, through the amount of days of this particular month (*N*_*MonthZ*_):

$$D_{CaseX}=\frac{Y_{MonthZ}}{N_{MonthZ}}$$ (3)

On days where the difference between *D*_*CaseX*_ and *C*_*CaseX*_ is negative, the shortfall has to be compensated by energy from the hydrogen system. We assume that if the yearly positive offsets, with the inclusion of the long-term storage efficiency in the hydrogen system of 38.3%, are high enough to outbalance the negative offsets, it is possible to rely fully on the energy produced in this year. Thus, the following inequality must hold true for a case to be considered self-sufficient with a realistic PV generation profile:

$$\sum D_{neg}\leq \sum D_{pos}*38.3\%$$ (4)

Where *D_neg_* are days where *D*_*CaseX*_ > *C*_*CaseX*_ and *D_pos_* are days where *D*_*CaseX*_ < *C*_*CaseX*_.

We also examine the sensitivity of results to both technical and cost uncertainties. The efficiency of PV modules is a key parameter due to the exclusive reliance on PV to generate electricity. We assume that our rooftop PV systems are sc-Si modules. Other work has shown that an efficiency of 27.2% can be reached by 2050 with a yearly increase by 0.3% [24]. We examine a range of PV efficiencies from the current efficiency of 17% to 27.2% [24, 25], focusing in particular on two cases: 22.1% (assuming an annual increase by 0.15% until 2050) and 27.2% (annual increase by 0.3%). According to Müller, Folini [77], the average yearly increase in efficiency of sc-Si cells over the last decade was 0.17%, so our assumptions are within a realistic range. We assume that CdTe thin-film modules are used for facade electricity production as they are more suited for difficult light conditions or when PV modules should be integrated into building parts with a heterogenous structure [23]. Efficiency of CdTe thin-film cells are assumed to be 0.787 times that of sc-Si cells, based on their relative efficiencies under standard conditions from [78]. Other technical parameters as detailed in the results below are varied while PV efficiency is kept at 27.2%. A final component of our sensitivity analyses are the cost of key components, to examine their effect on the economic feasibility of the self-sufficient cases. This includes costs in the alternative (fossil fuel and grid connected) cases. Their total costs are strongly influenced by the energy prices for electricity, gas, and gasoline. Finally, discount rate also affects total costs, so we vary it from its default of 4% to 0% and 6%.

## Results

### Feasibility of self-sufficiency

**Fig 2:**
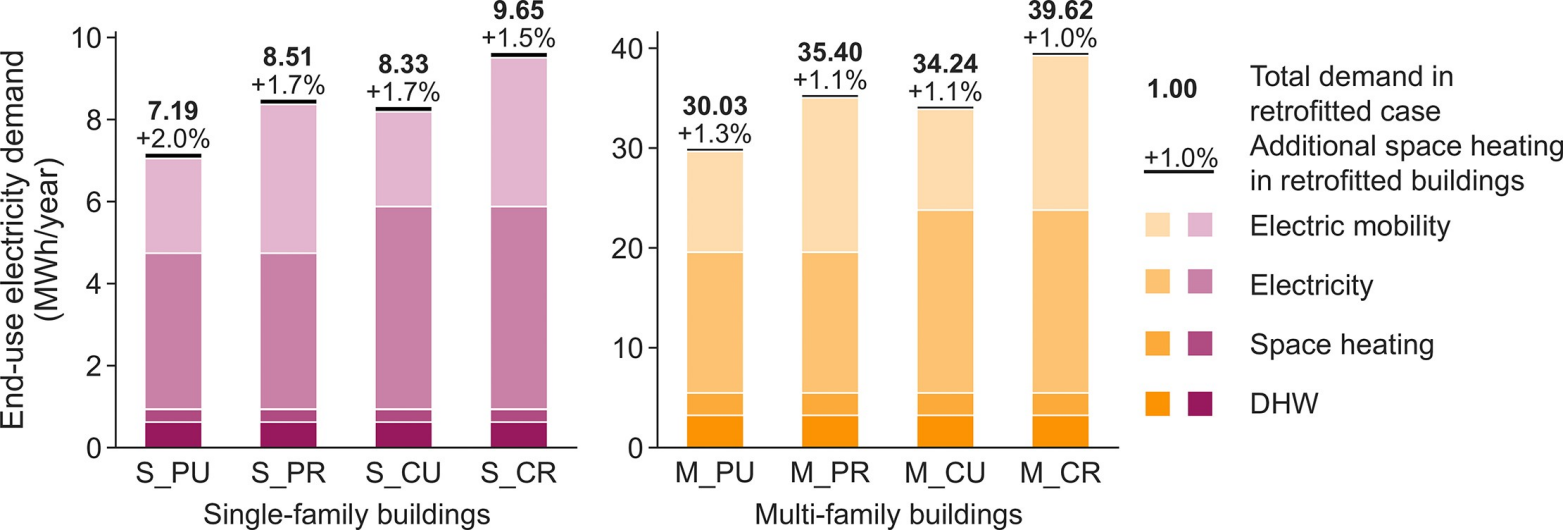
End-use electricity demand of each case in MWh/year. Retrofitted buildings require more energy than new buildings; this difference is indicated with the black bar and percentage difference on top of the total end-use electricity demand.

The composition of the end-use demand for electricity we use based on the assumptions laid out above is shown in Fig 2. It consists of domestic hot water (DHW), space heating, general electricity demand (for lighting, cooking, devices, etc.), and electricity for charging electric vehicles.

**Fig 3:**
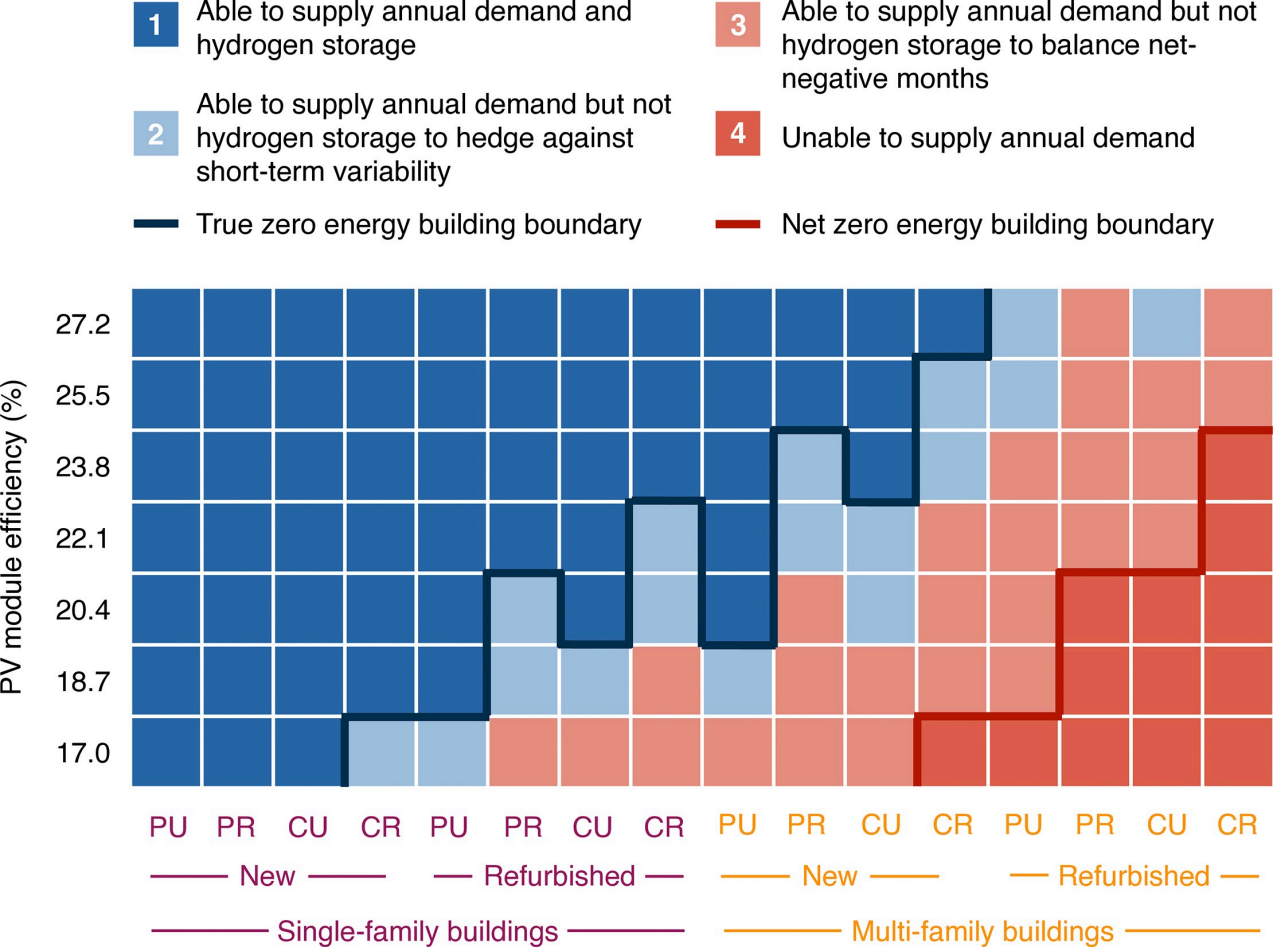
Feasibility of achieving true zero energy and net zero energy requirements as quantified by the energy balance level achieved, and as a function of the scenario and rooftop PV module efficiency. Only the last two identifying letters for each scenario are shown for increased readability, with **N**ew/**R**etrofitted and **S**ingle/**M**ulti-family building given by the lines spanning the x-axis below the scenario labels.

Based on this demand, and using the assumptions for technology deployment outlined in the methods above, Fig 3 shows the net energy balance as a function of the chosen scenario and the assumed PV module efficiency. All cases except for those shaded in dark red (marked 4 in the figure) can supply enough electricity over the entire year to meet annual demand and can thus be considered net zero-energy buildings. The net zero energy building boundary (dark red line) indicates this. In achieving net zero energy, only cases with MFBs show trouble at low PV efficiency values, but all cases are successful with high PV efficiencies. MFBs generally have more difficulties than SFBs because of their smaller available area for solar PV in relation to demand. If we add the condition that buildings must be able to supply additional electricity for hydrogen storage to use in net-negative months, more cases fail, indicated by the light red shading (marked 3). If we further tighten the conditions to demand hydrogen production be 1.75 times the amount determined to supply net-negative months in order to hedge against low solar yield for several days in a row, more cases fail, indicated by light blue shading (marked 2). This leaves the area shaded in dark blue (marked 1 in the figure): cases which are able to supply total annual demand as well as sufficient hydrogen production to balance net-negative months and shorter-term variability. These cases can thus be considered true zero energy buildings that could supply all of their inhabitants’ end-use electricity demand in a fully self-sufficient manner. The dark blue line marks this true zero energy building boundary in the figure.

**Fig 4:**
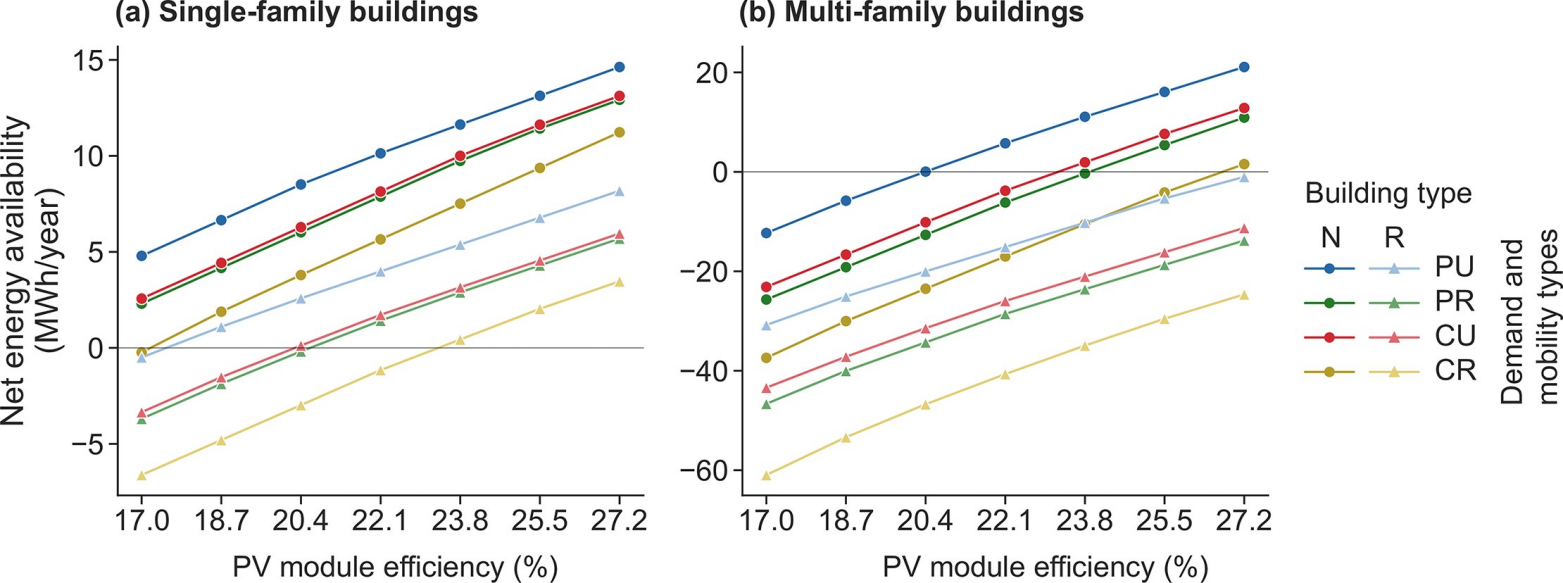
Cases with SFBs (left) and MFBs (right) considering backup hydrogen production in the size 1.75 times the optimized amount in net negative months. Values above zero net energy availability indicate cases that are successfully true zero energy buildings throughout the year. Values below this line indicate failed cases. The PV module efficiency is for rooftop PV systems; facade PV system efficiency tracks that of rooftop PV efficiency as indicated in the methods section above.

Fig 4 focuses on the feasibility of true zero energy buildings (i.e., our condition that 1.75 times the optimal amount of hydrogen can be produced). It shows how far above or below the balanced (supply equals demand) line the individual cases are, as a function of PV efficiency. Installed PV system capacities range for cases with SFBs from 12.7 kWp (SNPU, 27.2% rooftop PV efficiency) to 18.1 kWp (SRCR, 22.1% rooftop PV efficiency) while cases with MFBs require a capacity between 55.2 kWp (MNPU, 27.2% rooftop PV efficiency) and 77.4 kWp (MRCR, 22.1% rooftop PV efficiency). Because most cases include the use of facade PV, and the monthly share of solar energy production differs between rooftop PV and facade PV, installed capacities are not exactly equal for 22.1% and 27.2% efficiency. The discussion of PV system capacities leads us to the consideration of overall system dimensioning.

### System dimensioning for self-sufficiency

The battery, hydrogen, and PV system dimensions required for full self-sufficiency (true zero energy buildings) and assuming 27.2% rooftop PV efficiency are given in Table 5. Since the fuel cell and electrolyzer sizes are fixed (4.8 kW in SFBs and 9.7 kW in MFBs for electrolyzers, 2.1 kW in SFBs and 4.1 kW in MFBs for fuel cells) they are not shown in this table. The bottom four multi-family cases marked with a * are those which consider our design limits for allowed PV area (i.e., they are outside the true zero energy boundary in Fig 3).

**Table 5:** Battery and hydrogen tank sizes as well as area covered by the PV system per case. Required rooftop and facade area are given to fulfil the energy demand with the assumption of a rooftop PV efficiency of 27.2% and facade PV efficiency. Cases marked with * are considered to exceed our limits for PV area and thus not able to be fully self-sufficient.

	Battery size (kWh)	Hydrogen tank size (m^3^)	Rooftop PV area (m^2^)	Facade PV area (m^2^)
SNPU	38.6	9.0	46.7	0
SNPR	45.8	10.7	55.1	0
SNCU	44.9	10.4	54.2	0
SNCR	52.1	12.1	62.7	0
SRPU	39.4	9.2	48.2	0
SRPR	46.6	10.8	56.6	0
SRCU	45.7	10.6	55.7	0
SRCR	52.9	12.3	57.0	10.3
MNPU	162.5	37.8	200.0	0
MNPR	191.9	44.7	219.7	21.1
MNCU	185.6	43.2	219.7	11.7
MNCR	215.0	50.0	219.7	61.1
MRPU*	164.5	38.3	153.8	71.9
MRPR*	193.9	45.2	153.8	121.3
MRCU*	187.6	43.7	153.8	112.0
MRCR*	217.0	50.5	153.8	161.3

The table illustrates that the key factor causing lower feasibility of self-sufficiency for both retrofitted houses and multi-family houses is that of limited rooftop space for PV, relative to demand: the SRCR case is the only single-family case that needs to add facade PV to fulfill its design requirements. While we also assume that the additional demand in retrofitted buildings is higher than that in new buildings, which makes these cases more difficult to begin with, it is this area limitation which is the key design parameter.

**Fig 5:**
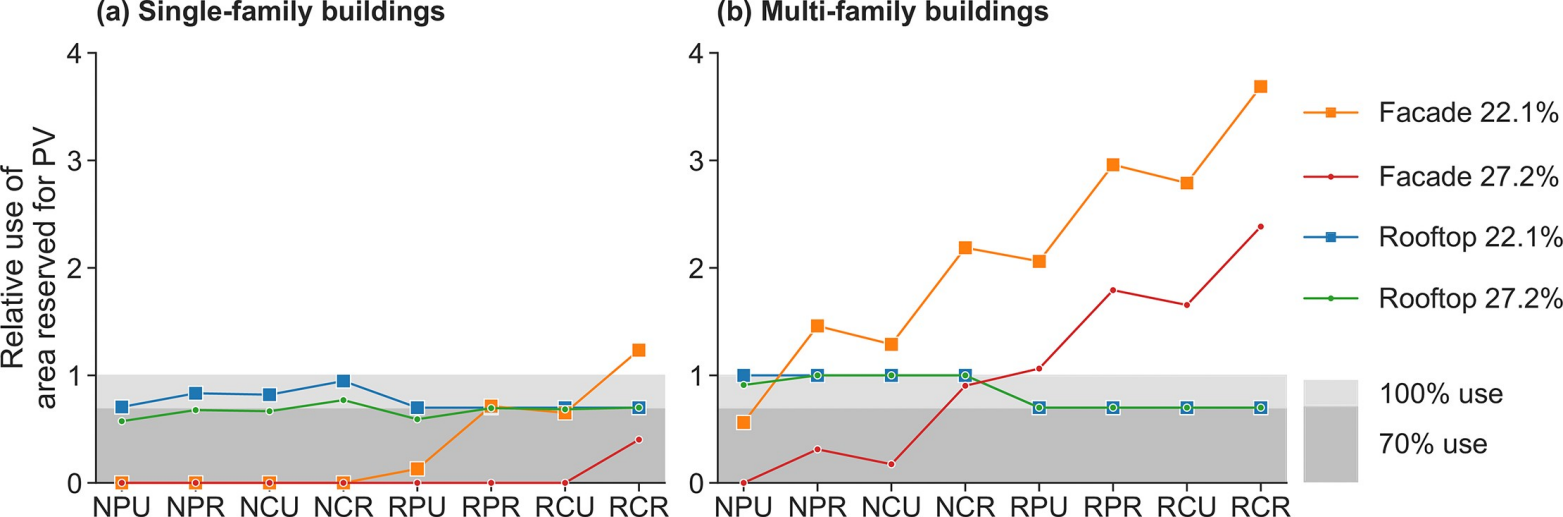
Relative use of rooftop and facade area per case with a rooftop PV efficiency of 22.1% and 27.2%, for (a) SFBs and (b) MFBs. The shaded 100% use border signalizes a full occupancy of the total available rooftop area. The shaded 70% use border signalizes full occupancy of the available rooftop area in retrofitted buildings, given that we assume those buildings have more pre-existing roof infrastructure which prevents 100% use.

We explore this in more detail in Fig 5, showing the relative share of the area we assume to be available used in each case as a function of scenario, and comparing 22.1% and 27.2% rooftop PV efficiencies. Rooftop area is always used first because of the higher efficiencies compared to facade PV. With an efficiency of 22.1%, the available facade area in SFB cases is sufficient, except for case SRCR, which can also be seen in Fig 3 where it is the only case outside the true zero energy building boundary at 22.1% efficiency. Where cases reach beyond the grey shaded region, they fail to fulfill our design requirements. However, they are not at their absolute maximum since we assume that only one third of one facade side is used for PV. Our results for the MFB cases require up to 3.7 times our designated facade area. Our facade use could be considered a conservative estimate. In most settings, however, substantially exceeding it is likely implausible, given buildings that were not designed for facade PV, and since we assume that average solar irradiation on all sides of the facade is 800 kWh/year. Nevertheless, it remains theoretically possible to consider two thirds of the sunniest facade side and one third each of less sunny facade sides covered; this would represent a relative use of 4 and would make true self-sufficiency technically possible in all of our cases.

### Costs for self-sufficient and alternative cases

**Fig 6:**
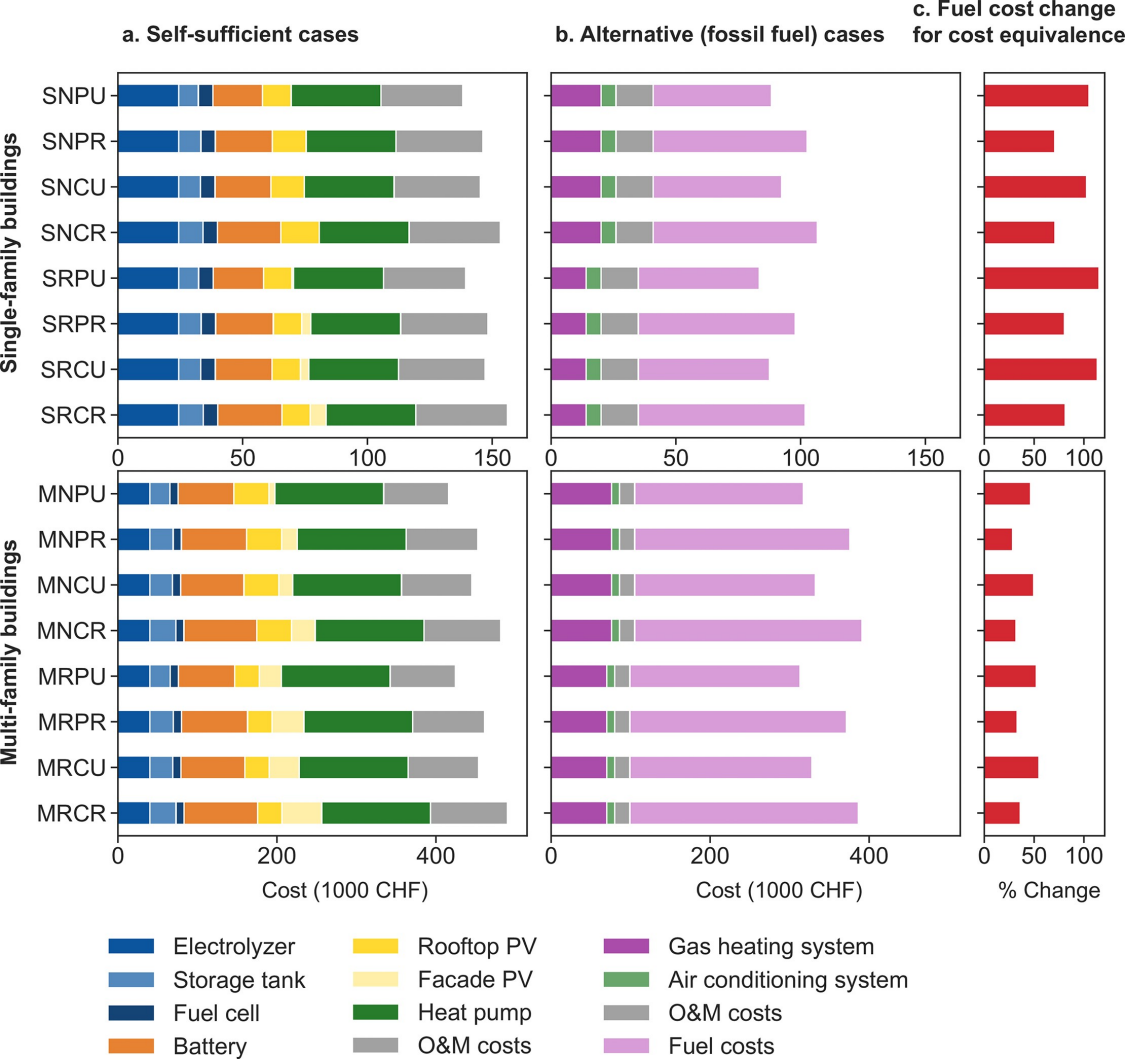
Share of total cost across different system components for (a) self-sufficient and (b) alternative cases, as well as (c) the percentage increase in fuel cost that would be required for alternative cases to become cost-equivalent with self-sufficient cases, assuming no other changes in component costs. All of these cost figures show total discounted costs over the entire lifetime of the systems, which is assumed to be 20 years.

The total discounted cost of the different components in each case is shown in Fig 6 for both self-sufficient and alternative cases. This cost data shown in the figure are for a rooftop PV efficiency of 22.1%. Rooftop and facade PV would get a marginally smaller cost if plotting 27.2%; but as the figure clearly shows, they are not the major cost components. The operation and maintenance costs in the self-sufficient cases are large; and on the same order as the investment cost for the PV system. These maintenance costs are mainly for the storage components (Table 3). Alternative cases assume no electrification of transport or heat; heating comes from gas burners, cooling comes from air conditioners (with electricity drawn from the grid), and transportation is by internal combustion engine vehicles. The fuel cost shown in the figure combines the cost of gas for heating and the cost of gasoline for driving.

**Fig 7:**
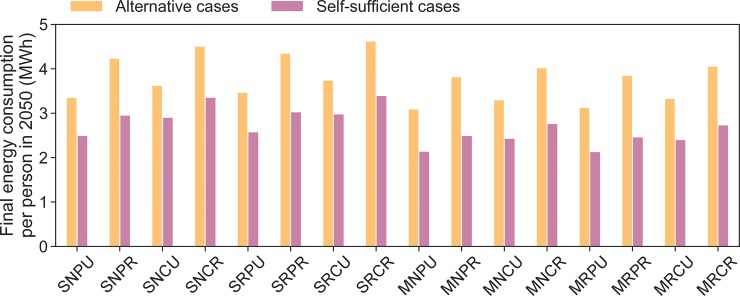
Required energy in alternative cases and produced energy in self-sufficient cases in 2050. Produced energy is provided with a rooftop PV efficiency of 27.2%.

As a comparison, we show (in Fig 6C) the fuel cost change that would be required for each alternative case to become cost-equivalent with the corresponding self-sufficient case, all other assumptions remaining equal. A 100% required fuel cost change means fuel costs would need to double. Cases with MFBs require a lower percentage increase of the prices than cases with SFBs, as the cost of the corresponding alternative cases have higher shares of energy prices. Another reason is that investment costs of technologies in MFBs are distributed across more useful energy demand met than in cases with SFBs. An important point that is not visible in Fig 6 is that self-sufficient cases require less energy in terms of their final energy consumption than alternative cases. Heat pumps and EVs are more efficient than gas heating systems and ICEs. Fig 7 shows this difference in final energy consumption of alternative and self-sufficient cases per person.

**Fig 8:**
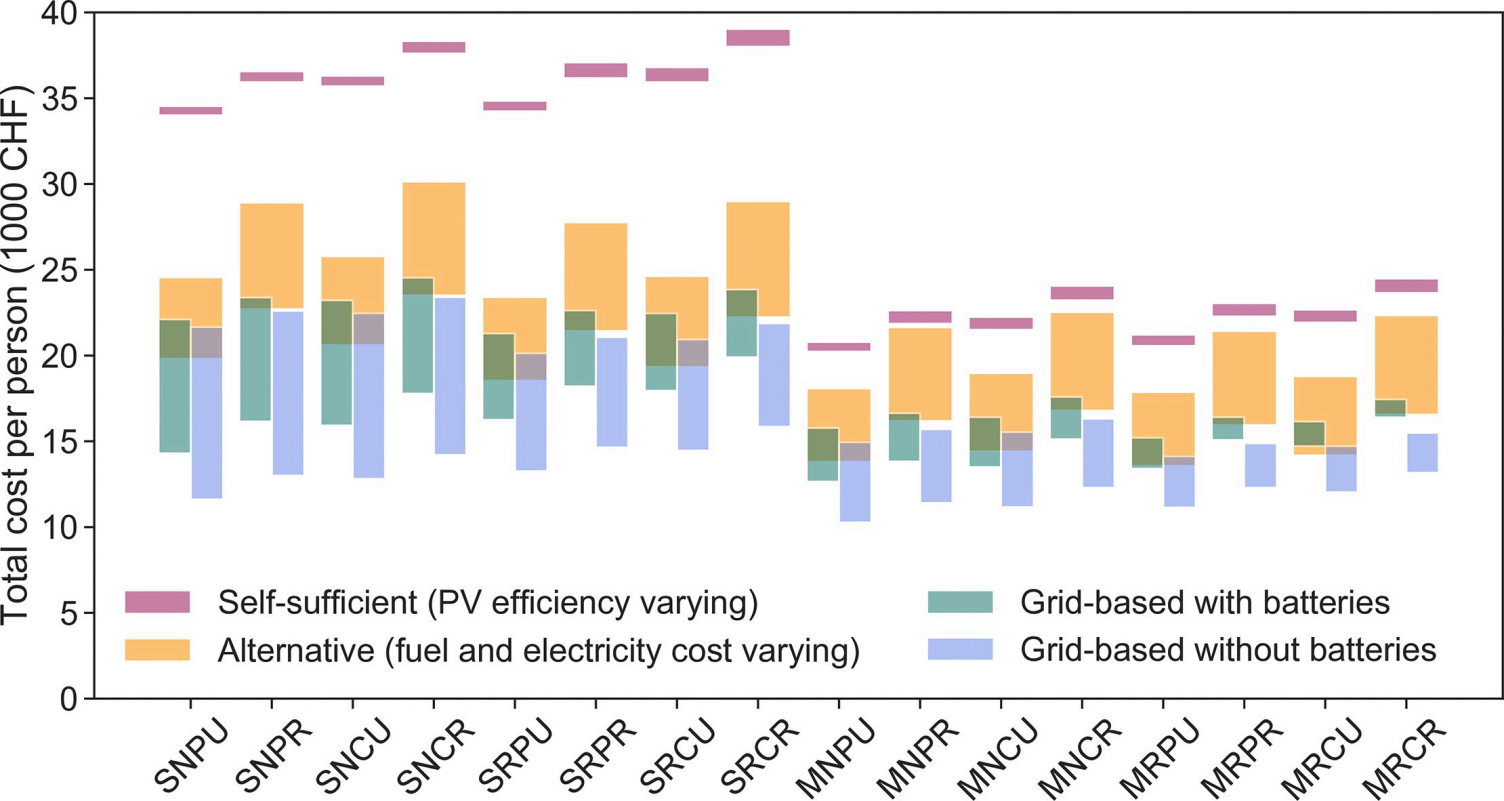
Total costs per person (4 people per building in SFBs, 20 people per building in MFBs). Self-sufficient cases show the range for PV efficiency varying between 22.1% and 27.2%. For sensitivity of self-sufficient cases to costs (including cost of the PV system), see Fig 11 below. Assuming battery cost of 265 CHF per kWh with a fixed additional 2,740 CHF for battery system setup. Alternative cases show costs assuming fuel, electricity, and gas costs ranging between + and– 20% of the assumptions in Table 4. The grid-based cases show range of costs assuming a selling price of electricity to the market of 0.16 CHF/kWh (scarce scenario) and 0 CHF/kWh (abundant scenario), and a purchasing price from the market of 0.31 CHF/kWh (scarce scenario) and 0.21 CHF/kWh (abundant scenario).

Total costs in the self-sufficient cases are generally higher than in alternative cases. However, as becomes clear in Fig 8, grid-connected fully electrified alternatives are almost always cheaper than fossil fuel alternative cases. In other words, unless electricity costs develop differently than assumed here, the most economically attractive option is full electrification while remaining connected to the grid. Grid-based cases with batteries are marginally, but not substantially more expensive; this is because we assume electricity can always be sold back to the local grid operator, so the battery brings little economic advantage. In reality, batteries would likely be desirable for distribution network balancing purposes. Moving from fully electrified but grid-connected houses to fully self-sufficient houses carries a major cost burden. Again, the cost of self-sufficiency per person is significantly lower in multi-family houses due to more efficient use of the installed infrastructure. In Fig 8, self-sufficient cases vary only marginally, based on PV efficiency varying from of 22.1% to 27.2%. Alternative cases have a larger range as the prices for energy were changed more drastically. The grid-based scenarios vary based on the scarce and abundant electricity supply scenarios. While generally, a conventional electricity demand and a driving behavior in a rural context result in the highest costs, the cost differences between different self-sufficient cases are comparatively narrow. As we will see further below in the sensitivity analyses, other factors such as varying discount rate have an even higher impact on overall cost, but this impact is the same across the separate scenarios.

Note that we assume no explicit subsidies whatsoever, in neither the self-sufficient nor alternative cases, with the exception of implicit subsidies embedded in fossil fuel costs [79]. Given the share of costs we see, the introduction of subsidies on storage technologies and/or heat pumps would appear most effective if a government wants to actively encourage self-sufficient households. On the other hand, terminating implicit fossil fuel subsidies or introducing levies on fossils fuels would decrease the relative attractiveness of the non-renewable cases (see Fig 6C).

### Sensitivity analyses

**Fig 9:**
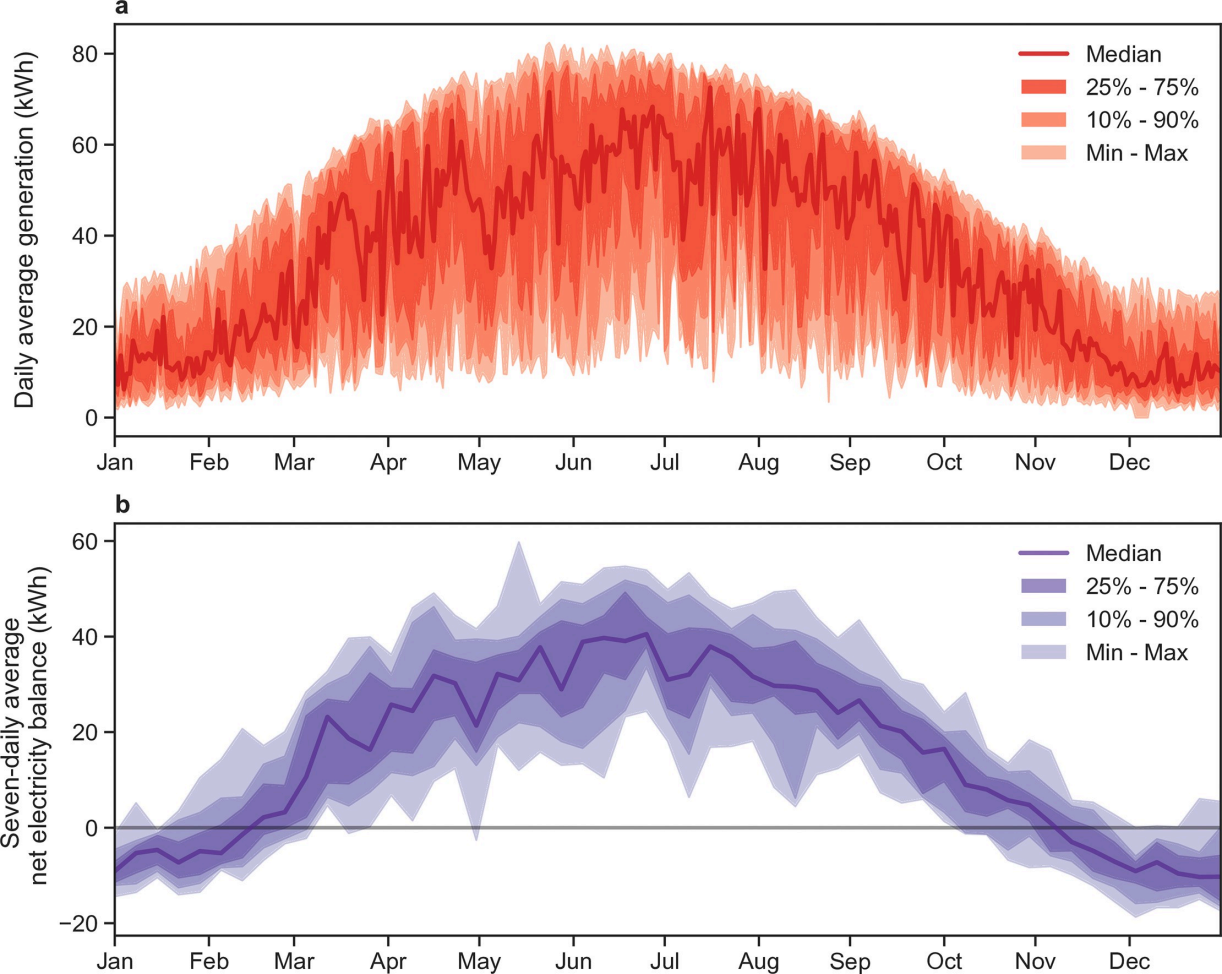
Variability of generation and net electricity balance the years 2000 to 2015 based on data for Worb, Bern, for the SNPU case **a.** Daily photovoltaic electricity generation. **b.** Seven-daily average net electricity balance.

To examine the sensitivity of results to a range of realistic solar irradiance profiles, we test all cases for each year in the period from 2000 to 2015 with daily data for the location of Worb, Bern, which we consider representative for a site in the Alpine foothills of Switzerland. Results show that the highest electricity production was in 2003 while the worst year was 2013, but in all cases, the overall energy balance is still positive (including full hydrogen production for self-sufficiency). Thus, we consider the results robust to year-on-year variability in PV system productivity. Exemplary for the SNPU case, and for all years 2000 to 2015, the variability of daily PV generation and the variability of weekly (seven-daily) net electricity balance considering electricity demand are shown in Fig 9 (see the optimized cases in S4 Data).

**Fig 10:**
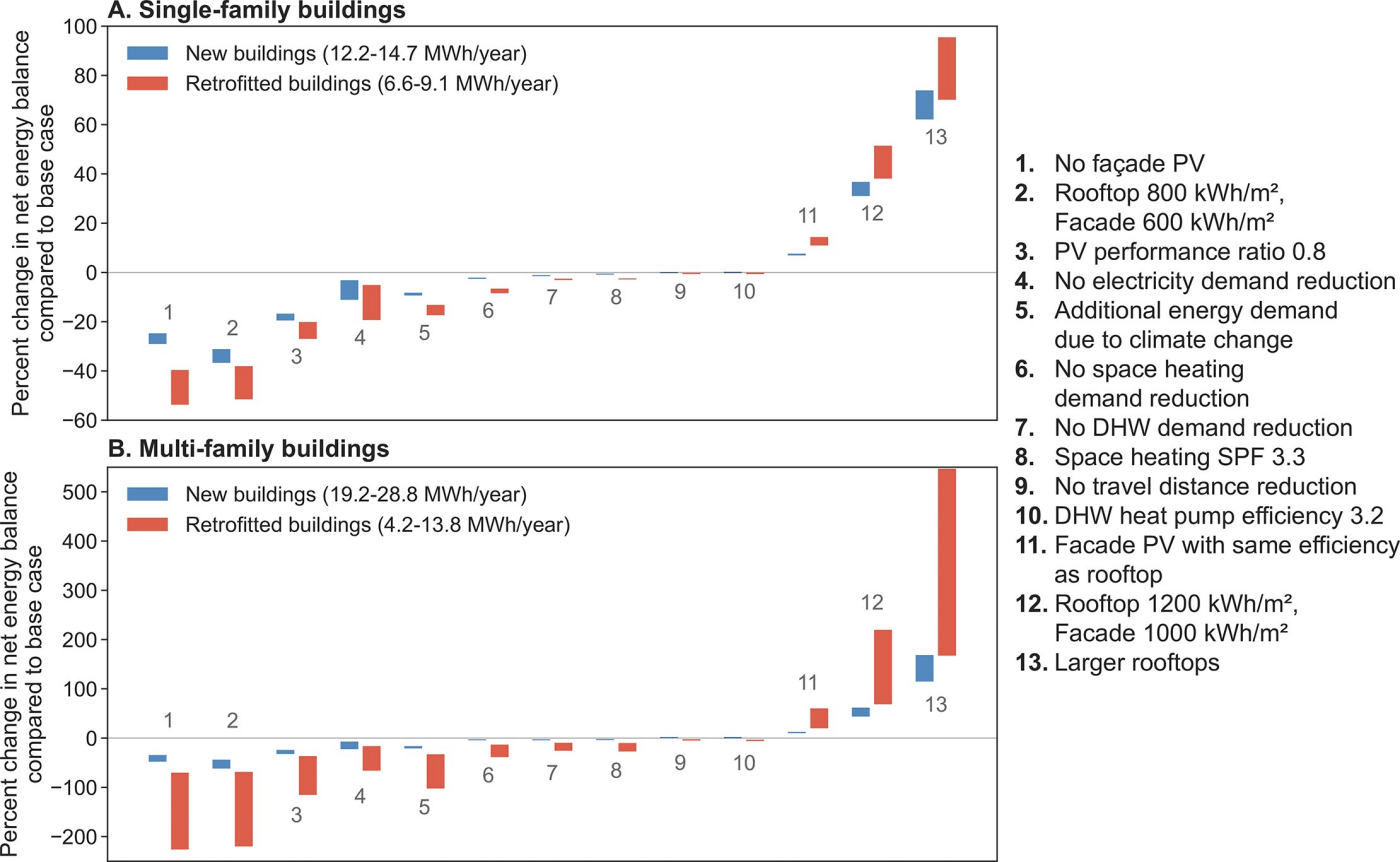
Technical sensitivity analysis for cases with SFBs (top) and MFBs (bottom). The bars show the range of changes across all rural-urban and conservative-profound scenario combinations, for either new (blue) or retrofitted (red) buildings. The base assumption in all cases is rooftop PV efficiency of 27.2% and no consideration of hydrogen production; the numbered adjustments are specific changes made to these assumptions. Additional energy demand due to climate change (adjustment number 5) is 25% of the median electricity demand, as defined by Nipkow (34). For larger rooftops (adjustment 13), we consider SFBs with only two stories but the same floor area, and MFBs with only three stories but the same floor area, resulting in an increase of the roof size of 50.0% on SFBs and of 66.6% on MFBs.

Results regarding the sensitivity of net energy balance to changing a range of technical assumptions are shown in Fig 10, assuming a rooftop PV efficiency of 27.2%, and no hydrogen production (i.e., fulfilling design parameters for net zero-energy buildings). This efficiency was chosen so that all net energy balances are positive for comparison. It is clear that irradiance and available area have the largest impact on the net energy balance (adjustments 1, 2, 12, and 13). Changes to the demand profile, including demand changes driven by climate change, have a lesser impact (adjustments 3, 4, 5, 11), or comparatively insignificant impact (adjustments 6, 7, 8, 9, 10). Retrofitted buildings deviate more than new buildings from the baseline assumptions because they are more affected by changes as they have a smaller available PV area and a slightly higher energy demand.

**Fig 11:**
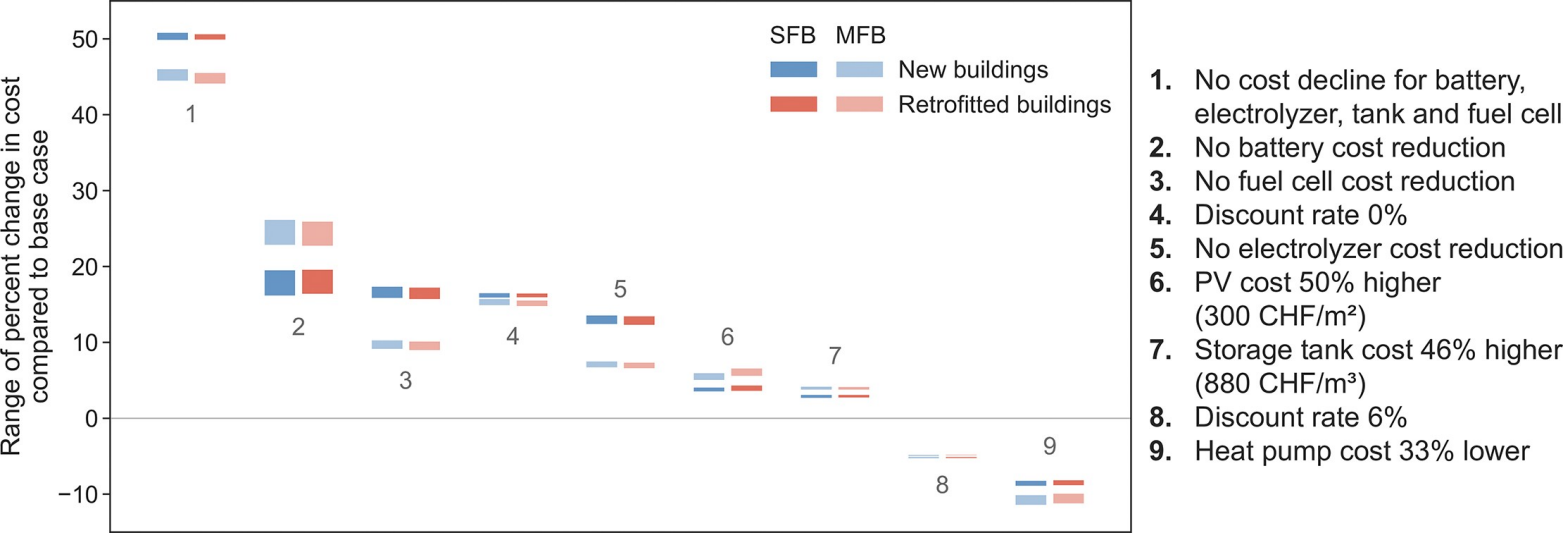
Cost sensitivity analysis of self-sufficient cases, showing the range of percent change in costs across all self-sufficient cases for each of the specific numbered adjustments made to the default cost assumptions, and differentiated between new/retrofitted buildings by color and between SFBs/MFBs by color intensity.

Results of the cost sensitivity analysis are depicted in Fig 11. The main sensitivity is to the cost of the storage components: batteries, fuel cell, electrolyzer and storage tank (adjustments 1, 2, 3, 5, 7 in the figure). The strong effect of battery costs is partly due to our assumption that they must be replaced after 15 years in operation, i.e. before end of the total system lifetime of 20 years, which increases expenditures. The higher storage tank price (adjustment 7) of 880 CHF/m^3^ instead of 600 CHF/m^3^ is based on a business-as-usual scenario by Marchenko and Solomin (71). A 50% higher PV cost only lets costs increase by 3.4–4.4% in SFB and 4.9–6.7% in MFB cases. In contrast, the discount rate has an effect ranging from –5.3% to +16.6% across all cases when varied from 0% to 6% (from its 4% base case value, adjustments 4 and 8 in the figure). We do not investigate lower than base case costs for the storage and PV components as the base case already assumes cost reductions from the current cost of these components. However, we investigate heat pumps at two thirds of the base case cost, finding that they would reduce overall costs by about 10%. Taken together, the technical and cost sensitivity analyses paint a clear picture. Technical feasibility, i.e. the net energy balance, is most affected by the ability to collect incoming sunlight as electricity, which in turn is influenced by available area and PV system performance. In contrast, economic feasibility, i.e. total system cost, is most affected by the cost of the various storage components of the system.

## Discussion and conclusion

We show that under a wide range of conditions, assuming full electrification of heating and transport, both single-family and multi-family buildings can be net zero energy buildings. In addition, a surprising number of cases are also able to function as true zero energy buildings and achieve full self-sufficiency. We also show that grid-connected and fully electrified cases remain substantially less expensive and are almost always less expensive than corresponding cases that retain the use of fossil fuels for heating and transportation. Thus, there is a clear case for full electrification, but there is a substantial premium to be paid for full self-sufficiency. The sensitivity analyses point out that the technical feasibility which our results show is robust to varying assumptions, including to the consideration of realistic PV generation profiles. Cost sensitivity analyses show that in absence of the expected cost declines in batteries, electrolyzers, tanks and fuel cells (i.e., all storage-related components), costs would be up to 50% higher. System costs are less sensitive to PV costs. This result underscores that the cost premium for self-sufficiency will depend primarily on the cost of the storage components that make self-sufficiency possible. The higher cost may not be prohibitive where self-sufficiency is desired for technical (e.g., remoteness) or social (e.g., desire for buildings perceived as ecologically friendly) reasons. The differences between SFB and MFB cases are interesting in two ways in particular. First, MFBs fail to achieve full self-sufficiency across a wider range of cases, simply because they have less area available for PV installations, relative to their electricity demand. However, MFBs are also substantially more cost-efficient on a per-person basis as investment into components is spread across more inhabitants. Thus, where MFB cases do achieve the potential for self-sufficiency, they ought to be economically more attractive.

The wide range of possible parameters to vary meant that assumptions for the creation of the self-sufficient cases needed to be restricted, for example, the assumption that only one third of one facade side is available, or the assumptions we made about the oversizing of hydrogen storage systems to address longer-term variability of PV generation. Based on our sensitivity analyses the results appear robust nevertheless, and we consider our assumptions to generally be conservative. A major uncertainty that we cannot fully address is the economic attractiveness of energy self-sufficiency by 2050. Under our base assumptions, it seems that grid-connected and fossil-powered cases have a cost advantage over self-sufficient cases. However, we cannot forecast 2050 prices, especially not for fuels. Furthermore, we know that continued reliance on fossil fuels is incompatible with preserving a stable global climate. Thus, it is highly likely that government intervention either through bans of or taxes on fossil fuels will change the price balance. Our analyses suggests that with even a relatively modest increase of fossil fuel costs, some of the self-sufficient scenarios become cost-competitive with grid-connected fossil-fired cases. Future work could also investigate the vulnerability of self-sufficient buildings to extreme weather conditions in more detail. By analyzing each weather year individually, we do not transfer stored hydrogen at end-of-year to the following year; this would result in additional supply security in reality. However, the effect of long dark winter periods could also be more substantial in particularly bad years, given our coverage of weather only from 2000–2015 occur that one year in the future has a worse solar yield than the worst year in the analyzed period. In such cases, hydrogen would have to be purchased on the market or hydrogen backup system size would have to be further increased. Additional work is also needed on performing full environmental impact assessments of self-sufficiency including all upstream energy and material use for different cases, as well as modeling cases located in other destinations worldwide with adjusting the data. Finally, we do not model optimal control of EV charging in combination with building-integrated storage components–implementing smart technologies for this may well reduce the required size of batteries and hydrogen storage in buildings, and thus bring costs down substantially.

Overall, we find that self-sufficient households are technically feasible across a wide range of scenario combinations. Their financial attractiveness depends on a variety of costs, particularly those of storage components, as well as on discount rates and primary energy prices by 2050. Given the perceived attractiveness of completely energy self-sufficient households, it is likely that more such buildings will be constructed even if fully electrified but grid-connected households are substantially less expensive. Falling costs of storage and political will to push for self-sufficiency may well in combination result in such buildings become more common than currently expected.

## Supporting information

S1 Dataset Definitions of cases and technological assumptions. File name S1_Methods_TechPotential_And_AltCases.
(XLSX)

S2 Dataset Definition of cases with hydrogen production to fulfill requirements of a true zero energy building. File name S2_Methods_Scenarios_Baseline_Optimized.
(XLSX)

S3 Dataset Definition of cases with a hydrogen production 1.75 times the amount determined to supply net-negative months. File name S3_Methods_Scenarios_BackupStorage175.
(XLSX)

S4 Dataset Definition of cases which are connected to the grid and still use batteries for short-term storage. File name S4_Methods_GridBased_withBatteries.
(XLSX)

S5 Dataset Definition of cases which are connected to the grid but do not use batteries for short-term storage. File name S5_Methods_GridBased_withoutBatteries.
(XLSX)
